# Supplementary material for: Conditional chemoconnectomics (cCCTomics) as a strategy for efficient and conditional targeting of chemical transmission
Source: eLife. 2024 Apr 30;12:RP91927. doi: 10.7554/eLife.91927 (PMC11060718; doi:10.7554/eLife.91927)
Supplement: Figure 3—source data 3. [file elife-91927-fig3-data3.zip › Figure 3 source data3 part 1.zip!/Figure 3-Source data 3-part1/1063f3-1-CRISPResso_Report_kboyr13q.zip!/CRISPResso_on_1063f3_4/9.Alleles_frequency_table_around_sgRNA_aatcacaccagtgggagcac.pdf]

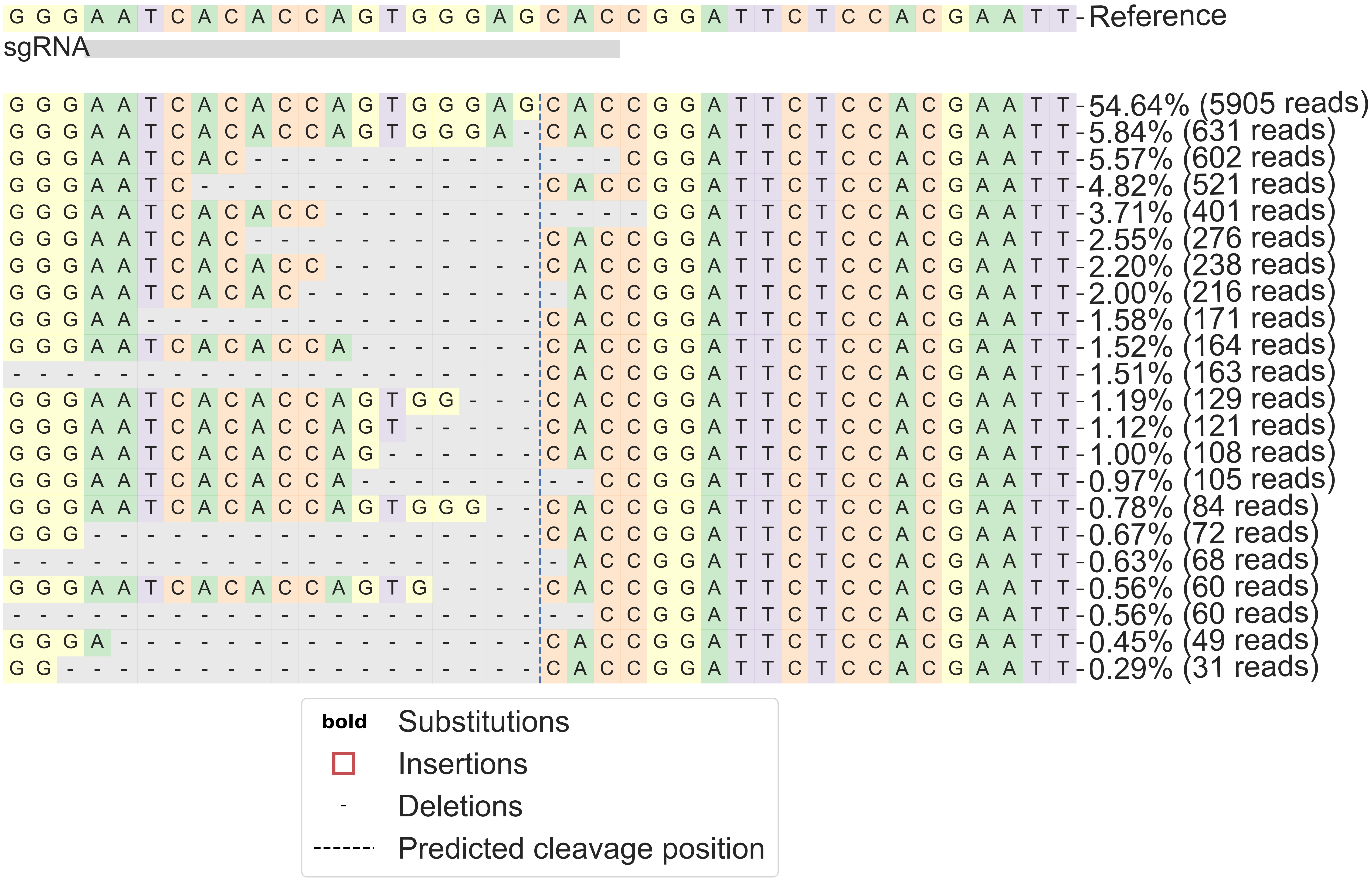

GGGAATCACACCAGTGGGAGCACCGGATTCTCCACGAATT Reference
sgRNA
GGGAATCACACCAGTGGGAGCACCGGATTCTCCACGAATT 54.64% (5905 reads)
GGGAATCACACCAGTGGGA-CACCGGATTCTCCACGAATT 5.84% (631 reads)
GGGAATCAC--------------CGGATTCTCCACGAATT 5.57% (602 reads)
GGGAATC-------------CACCGGATTCTCCACGAATT 4.82% (521 reads)
GGGAATCACACC------------GGATTCTCCACGAATT 3.71% (401 reads)
GGGAATCAC-----------CACCGGATTCTCCACGAATT 2.55% (276 reads)
GGGAATCACACC--------CACCGGATTCTCCACGAATT 2.20% (238 reads)
GGGAATCACAC----------ACCGGATTCTCCACGAATT 2.00% (216 reads)
GGGAA---------------CACCGGATTCTCCACGAATT 1.58% (171 reads)
GGGAATCACACCA-------CACCGGATTCTCCACGAATT 1.52% (164 reads)
--------------------CACCGGATTCTCCACGAATT 1.51% (163 reads)
GGGAATCACACCAGTGG---CACCGGATTCTCCACGAATT 1.19% (129 reads)
GGGAATCACACCAGT-----CACCGGATTCTCCACGAATT 1.12% (121 reads)
GGGAATCACACCAG------CACCGGATTCTCCACGAATT 1.00% (108 reads)
GGGAATCACACCA---------CCGGATTCTCCACGAATT 0.97% (105 reads)
GGGAATCACACCAGTGGG--CACCGGATTCTCCACGAATT 0.78% (84 reads)
GGG-----------------CACCGGATTCTCCACGAATT 0.67% (72 reads)
---------------------ACCGGATTCTCCACGAATT 0.63% (68 reads)
GGGAATCACACCAGTG----CACCGGATTCTCCACGAATT 0.56% (60 reads)
----------------------CCGGATTCTCCACGAATT 0.56% (60 reads)
GGGA----------------CACCGGATTCTCCACGAATT 0.45% (49 reads)
GG------------------CACCGGATTCTCCACGAATT 0.29% (31 reads)
bold Substitutions
Insertions
- Deletions
Predicted cleavage position
